# Validation and performance of a geriatric early warning score (GEWS) versus the national early warning score (NEWS) in predicting clinical deterioration in frail older patients

**DOI:** 10.1007/s41999-025-01316-7

**Published:** 2025-10-06

**Authors:** H. Baeyens, F. Haegdorens, S. Martens, M. E. Vanden Abeele, S. Wallaert, N. Van Den Noortgate, A. D. H. Brys

**Affiliations:** 1 Department of Geriatrics, Faculty of Medicine and Health Science, Academic Consultant Ghent University and KU Leuven, Staff Member Health Policy at Solidaris Health Insurance, Brussels, Belgium; 2 Nurse and Midwifery Sciences, Centre for Research and Innovation in Care (CRIC), Workforce Management and Outcome Research in Care Group, Faculty of Medicine and Health Science, University of Antwerp, Antwerp, Belgium; 3 Department of Geriatrics, St Trudo Hospital, Sint-Truiden, Belgium; 4 Department of Geriatrics, Jessa Hospital, Hasselt, Belgium; 5 Biostatistics Unit, Faculty of Medicine and Health Sciences, Ghent University, Ghent, Belgium; 6 Department of Geriatrics, Ghent University Hospital, 9000 Ghent, Belgium

**Keywords:** EWS, GEWS, Frail, Older people, Validation, Clinical burden

## Abstract

**Purpose:**

A Geriatric Early Warning Score (GEWS) for detecting clinical deterioration in frail older individuals was developed to account for age-related altered physiological responses and was based on the framework of the National Early Warning Score (NEWS), expert consensus and existing literature. This study aimed to validate GEWS and compare its predictive accuracy with the NEWS, as well as to evaluate the clinical burden of predefined thresholds.

**Methods:**

In this prospective multicenter observational study, patients receiving acute geriatric care were included. Clinical deterioration was defined as: (1) unexpected death, (2) Intensive Care Unit transfer, (3) transition to palliative care, or (4) urgent medical/surgical intervention.

**Results:**

Among 511 patients, 348 events occurred in 297 individuals. We found higher AUROC 0.796 [95% CI 0.770–0.822] and PR-AUC values 0.412 [95% CI 0.350–0.472] for GEWS compared to NEWS 0.732 [95% CI 0.697–0.765] and 0.305 [95% CI 0.249–0.365] respectively (*p* < 0.0001). The GEWS ≥ 5 compared to NEWS ≥ 5 was superior in accuracy (0.940 vs. 0.927), PPV (0.497 vs. 0.365), and specificity (0.977 vs. 0.967), (all *p* < 0.0001). Moreover, a lower-number-needed-to-evaluate (NNE) (2.013 vs. 2.738, *p* < 0.0001) and a higher alerted-outcome-event-rate (AOER 4.424 vs. 3.706, p < 0.0005) resulted in lower clinical burden. For life-threatening events (Type 1–3), GEWS ≥ 8 compared to NEWS ≥ 7 showed higher specificity (0.999 vs. 0.995, *p* < 0.0001) and reduced rate-of-alerts (ROA 0.518 vs.1.347, *p* < 0.0001).

**Conclusion:**

GEWS more accurately detects clinical deterioration within 12 h in frail older adults than NEWS, with less clinical burden. GEWS ≥ 5 is recommended for clinical alerting, whereas ≥ 8 for more urgent medical assistance.

**Supplementary Information:**

The online version contains supplementary material available at 10.1007/s41999-025-01316-7.

## Key summary points

**Aim:**

Validation and performance of a Geriatric Early Warning Score (GEWS) in frail older patients.

**Findings:**

GEWS is a more accurate and geriatric-specific alternative to the National Early Warning Score (NEWS) for detecting clinical deterioration in frail older patients. A GEWS ≥ 5 offers the best balance between predictive accuracy and clinical burden, making it the preferred threshold for routine monitoring. GEWS ≥ 8 is recommended for high-level urgent medical assistance.

**Message:**

GEWS more accurately detects clinical deterioration within 12 h in frail older adults than NEWS, with less clinical burden for healthcare providers.

**Supplementary Information:**

The online version contains supplementary material available at 10.1007/s41999-025-01316-7.

## Introduction

The use of an Early Warning Score (EWS) is recommended to reduce preventable in-hospital mortality by facilitating early detection of patient deterioration. An EWS is a composite score derived from deviations in multiple vital parameters, designed to alert healthcare providers to physiological decline that may precede severe adverse events. Nurses calculate EWSs during routine patient assessments, enabling timely escalation of care and potentially preventing critical deterioration [1].

Since its introduction, various EWSs have been developed, incorporating different thresholds for escalation, a range of vital parameters, and specific adaptations for selected populations, such as patients with chronic obstructive pulmonary disease (COPD), children and pregnant women [2–4]. In current hospital practice, older patients are typically assessed using the National Early Warning Score (NEWS), its updated version NEWS2 [1, 5] or the Modified Early Warning Score (MEWS) [6].

However, increasing evidence indicates that these standard EWSs may underperform in older patients. Age-related physiological and pathophysiological changes can alter the thresholds at which vital parameters become clinically meaningful [7], thereby limiting the predictive accuracy of these tools in the older population. For instance, Churpek et al. [8] demonstrated reduced predictive accuracy of MEWS in patients over 65, while Rønningen et al. [9] reported poor prognostic performance of NEWS2 in frail patients over 70 hospitalized with COVID-19. In response, Vardy et al. [10] proposed supplementing NEWS2 with the Clinical Frailty Scale (CFS) and the four-A-test (4AT) for delirium screening. More recently, Nissen et al. combined NEWS with age to improve in-hospital mortality prediction in older emergency department (ED) patients, and later developed the Frailty-adjusted Prognosis in the ED (FaP-ED) tool, by integrating NEWS with CFS [11, 12]. Similarly, Candel et al. [13], introduced the International EWS (IEWS), which incorporates NEWS, age and sex.

In Belgium, the care of older patients with frailty is primarily managed by geriatricians. Approximately 90–95% of the patients hospitalized in acute geriatric wards are admitted via the ED, with the remainder mostly transferred from other hospital units. They typically present with acute medical conditions, often accompanied by significant frailty and multimorbidity [14]. Decisions to escalate care are often based on individual vital parameters and clinical intuition. While some wards use local ‘track and trigger’ systems or have implemented NEWS within electronic patient records (EPR), practice varies considerably between hospitals.

Importantly, qualitative research by Geeraert et al. [15] among Flemish geriatricians revealed concern about the use of NEWS in this patient population, generating excessive false positive alarms that increase clinical workload alongside false negative alarms that compromise patient safety. Their findings underscored the need for a geriatric-specific EWS accurate in detecting clinical deterioration and avoiding inappropriate clinical burden.

To address this gap, later confirmed by the aforementioned literature, the Geriatric Early Warning Score (GEWS) was developed in 2018 for acute care of frail older patients, through expert consensus among Flemish geriatricians (Az Alma Hospital), guided by existing literature on on age-specific deviations in vital parameters among frail older adults. A pilot study [16] confirmed the clinical feasibility of GEWS, but formal validation to assess the predictive accuracy for short-term (12-h) outcomes most relevant in geriatric care as unexpected death, Intensive Care Unit (ICU) transfer, and transition to palliative care, was still needed. We decided to include a fourth outcome for acute clinical deterioration requiring urgent medical or surgical intervention, enabling timely stabilization, avoid inappropriate care and initiating shared decision-making about care preferences.

### Study aims

First, we wanted to validate GEWS compared to NEWS as gold standard by evaluating its predictive accuracy for the four predefined outcomes within a 12-h period in hospitalized geriatric patients with frailty.

The secondary objective was to evaluate predefined GEWS thresholds considering clinical burden. To this end, we calculated the number-needed-to-evaluate (NNE) and the rate-of-alerts (ROA). This dual focus was designed to identify the GEWS threshold that provides the best balance between predictive performance and clinical feasibility, both at the ED and on geriatric wards.

## Subjects and methods

### Study population and design

This multicenter, prospective observational study was conducted between September 1, 2022, and February 28, 2023, in the acute geriatric wards of three Belgian teaching hospitals: Az Alma, St. Trudo and Jessa Hospital. All patients admitted to these geriatric wards with a CFS ≥ 3 were eligible for inclusion. Exclusion criteria were patients with terminal or palliative care needs at admission and patients undergoing elective surgery. Written informed consent was obtained by the attending geriatrician from either the patient or their designated caregiver. The study protocol was approved by the medical ethics committee of the participating hospitals of Jessa Hospital, Hasselt (registration number: B2432022000020).

### Data collection

#### Measurement instruments

The NEWS, developed by the Royal College of Physicians, assigns scores from 0 to 3 to deviations in six key vital parameters, depending on the degree of abnormality (Table 1a). This composite score is used to monitor patient status and prompt escalation of care.

**Table 1:** The National Early Warning score (NEWS) and the Geriatric Early Warning Score (GEWS),

	NEWS[5]						
(a)							
NEWS Score	3	2	1	0	1	2	3
Respiration rate (p/m)	≤ 8		9–11	12–20		21–24	≥ 25
Oxygen saturation (%)	≤ 91	92–93	94–95	≥ 96			
Oxygen therapy		Yes		No			
Body temperature (°C)^1^	≤ 35.0		35.1–36.0	36.1–38.0	38.1–39.0	≥ 39.1	
SBP (mmHg)	≤ 90	91–100	101–110	111–219			≥ 220
Heart rate (bpm)	≤ 40		41–50	51–90	91–110	111–130	≥ 130
Level of consciousness (AVPU)				Alert			Voice stimulus Pain stimulus Unresponsive

	GEWS[16]						
(b)							
GEWS- score	3	2	1	0	1	2	3
Respiration rate (p/m)	< 12			12–23	24–27		≥ 28
Oxygen saturation (%)	< 86	86–88	89–90	91–100			
Oxygen therapy (L/min)	≥5	4–2	1	0			
Body temperature (°C)^1^	< 35.0			35.0–37.1	37.2–37.4	37.5–38.0	> 38.0
SBP (mmHg)	< 100	100–109	110–119	120–189		190–220	> 220
Heart rate (bpm)	< 50			50–75	76–95	96–120	> 120
Level of consciousness (AVPU)	Unresponsive	Pain stimulus	Voice stimulus	Alert			Agitation
Pain (NRS/PAINAD)				0	1–3	4–6	≥ 7

p/m: per minute; %: percentage; °C: degree Celsius,^1^temperature measured at the axilla, temporal or tympanic sites. For forehead measurements, 0.2 °C was added to the recorded value; SBP: systolic blood pressure; mmHg: millimeters of mercury; bpm: beats per minute; NRS: Numeric rating scale (ranging from 0 to 10); PAINAD: Pain in advanced dementia scale (ranging from 0 to 10)

The GEWS was developed using the NEWS framework. Its design was based on expert consensus, supported by existing literature on age-related physiological responses and clinical deterioration patterns in older adults [7, 16–18]. GEWS clinical feasibility was tested in a pilot study[16]. As shown in Table 1b, GEWS introduces four key adaptations to NEWS. First, the cut-off values for vital parameters were adjusted to better account for the frailty of older adults. Frailty reduces physiological reserves to respond to external stressors, as such even minor deviations in vital parameters can lead to severe outcomes. Consequently, GEWS features narrower cut-off ranges of vital parameters compared to NEWS. Second, GEWS quantifies the amount of oxygen therapy more precisely, whereas NEWS only employes a binary classification (oxygen therapy yes/no). By experience we assumed that a higher need of oxygen would reflect higher disease severity. This was confirmed by a meta-analysis in 2024 that concluded that graded oxygen models improve recognition of deterioration [19]. Third, the scoring system for assessing consciousness in patients with delirium, whose mental status can fluctuate over time, was refined. In the original NEWS, agitated patients are typically scored as ‘alert’, potentially leading to missed delirium diagnoses. NEWS2 addressed this by introducing the category of ‘new confusion’ as the highest score; however, this adjustment achieved only 4.3% sensitivity, compared to 20.0% when using the 4AT-score[20]. GEWS further improves upon this by assigning the highest score to ‘agitation’ and implementing a more gradual scale for other levels of consciousness, enhancing the detection of delirium-related fluctuations. Finally, GEWS incorporates pain as a sixth vital parameter, assessed using the Numeric Rating Scale (NRS) or the Pain in Advanced Dementia scale (PAINAD). This ensures effective pain monitoring, even in patients with limited communication abilities, addressing a clinical gap where pain – an early indicator of acute medical events – is often overlooked in this patient population. Additionally, the presence of pain can increase cardiovascular stress, potentially leading to a faster decline in patients with multiple comorbidities and less pathophysiological reserve.

#### Predefined EWS thresholds

Because NEWS and GEWS differ in both the parameters included and their cut-off scores, the thresholds for clinical escalation can differ. For NEWS, a threshold of ≥ 5 or any single vital parameter scoring 3 (NEWS ≥ 5 S3) is widely used in clinical practice as the point at which to escalate care [5]. For GEWS, a threshold of ≥ 4 or any single parameter scoring 3 (GEWS ≥ 4 S3), was predefined based on expert consensus and clinical relevance. This threshold was chosen to reflect either a moderate deviation in at least two parameters (each scoring 2 points) or a severe abnormality in one parameter (scoring 3 points). This is aligned with clinical management orders used in our clinical practice. For triggering an urgent medical assistance call, a NEWS threshold of ≥ 7 is recommended [5]. In GEWS, a corresponding threshold of ≥ 8 was predefined, post-hoc based on findings from our prior feasibility pilot study [16] and reflects a high-risk state requiring urgent intervention.

#### Baseline clinical data

The following baseline information was retrospectively retrieved from the EPR: age, sex, CFS assessed by the geriatrician, delirium diagnosis at the time of a clinical deterioration event (assessed with the Confusion Assessment Method (CAM) or Delirium Observation Scale (DOS) by the nurses), Do-Not-Resuscitate (DNR) status at admission and length of hospital stay.

### Study procedure

After obtaining written informed consent, participants were enrolled in the study. Vital parameters included temperature, respiratory rate, oxygen saturation, oxygen therapy (l/min), pulse rate, systolic blood pressure, pain score (NRS or PAINAD), and level of consciousness (Alert, Voice, Pain, Unresponsive (AVPU), or agitated).

Vital parameters were measured upon arrival at the ED, throughout patients’ stay in the ED and prior to discharge from the ED. Once upon arrival at the geriatric ward, vital parameters were measured at admission and subsequently by nursing staff during routine morning and afternoon shifts and manually entered into the EPR. Nurses alerted the physician based on the patient’s condition, specific geriatric instructions or their clinical intuition. Clinical deterioration events that needed urgent intervention were prospectively recorded by the attending geriatrician including precise timestamps (year/month/day, hour/minutes/seconds), both at admission and throughout hospitalization.

Although the attending geriatricians also served as study investigators, potential bias was minimized, as the NEWS and GEWS scores were not available during clinical care. These scores were calculated retrospectively after the study period, based on data exported from the EPR, and therefore did not influence any treatment decisions. All data were automatically extracted from the EPR after study completion and subsequently linked to patient characteristics and clinical deterioration event records. All data were pseudonymized and then centralized by the principal investigator. Only the principal investigator and geriatrician (H.B.) was engaged in processing the exported data for research purposes.

### Outcome classification

Clinical deterioration was defined as the occurrence of one of four clinical events, categorized as follows:

**Table:** 

Type 1 Event	Unexpected death
Type 2 Event	Unanticipated intensive care transfer (i.e. stroke unit, coronary care unit, ICU or medium care unit)
Type 3 Event	Unexpected transition to palliative care following an acute, unexpected event
Type 4 Event	**Urgent, unexpected medical intervention within 12 h, including**: • **Consciousness related interventions**: ▪ Intravenous (IV) glucose (> 6 g) for acute hypoglycemic coma • **Cardiovascular interventions**: ▪ IV diuretics (for acute pulmonary oedema) ▪ IV or oral anti-arrhythmics (for life-threatening arrhythmia) ▪ IV nitrates (for unstable angina pectoris) • **Bleeding or hemostasis interventions**: ▪ Transfusion of packed red blood cells, platelets (for massive hemorrhage) ▪ Synthetic plasma factors (for massive hemorrhage) ▪ Anticoagulants (e.g. low-molecular-weight heparin (LMWH)), heparin, or thrombolytics (for embolism, stroke or myocardial infarction) • **Sepsis and dehydration related intervention:** ▪ Volume resuscitation with fluid challenge (crystalloids/electrolytes/albumin) ▪ IV or oral antibiotics ▪ IV corticosteroids (for acute Addison’s disease) • **Respiratory interventions:** ▪ Oxygen therapy ▪ IV corticosteroids (for COPD exacerbation) **Urgent unexpected surgical intervention within 12 h**

Type 4 events served as a proxy outcome to indicate the presence or absence of acute clinical deterioration. This classification was not intended to represent varying levels of acuity, but rather to capture any unanticipated medical intervention initiated by a geriatrician in response to a perceived clinical deterioration. Each listed intervention required a geriatrician’s assessment and decision, ensuring that all events reflected a meaningful change in the patient’s condition.

### Data analysis

We divided the hospitalization period into two distinct episodes to ensure a sufficient number of events occurring during hospitalization and having enough Type 1–3 events. Typically, patients were admitted to the ED with a Type 4 event, whereas Type 1–3 events tended to occur later in the hospitalization. Each episode could include a Type 4 event, but only one Type 1–3 event per patient was allowed.

The first episode started at admission to the ED (or to the geriatric ward in case the patient was transferred from another hospital ward) and lasted for at least 72 h, provided that no event occurred within these first 72 h. If one or more Type 4 events occurred within the first 72 h, the second episode started 72 h after the last Type 4 event of the first episode and continued until the patient was discharged from the geriatric ward. The choice of a 72-h window was based on the findings by Subbe et al. [6], who demonstrated that only 1.8% of patients continued to present with critical vital parameters related to the initial reason for admission beyond 72 h.

The unit of analysis was a single observation set of vital parameters. Observation sets recorded within 12 h before a clinical event were categorized as *event observation sets*. Observation sets recorded more than 24 h before an event were classified as *non-event observation sets*. Observation sets recorded between 24 and 12 h before an event and observation sets recorded after an event, were categorized as *neutralized observation sets*. To minimize treatment bias, all neutralized observation sets were excluded from the analysis. As a consequence, after the occurrence of a Type 1–3 event, all subsequent observation sets were categorized as *neutralized observation sets*. In analogy, after occurrence of a Type 4 event, all subsequent observation sets were categorized as *neutralized observation sets* until the end of the respective first or second episode. However, if a Type 1–3 event occurred within 12 h of a preceding Type 4 event, both were merged and categorized as a single Type 1–3 event. In contrast, if a Type 1–3 event occurred more than 12 h after a preceding Type 4 event, they were considered as two distinct events. Only in the latter case, observation sets recorded after a Type 4 event were categorized as *event observation set* and were not neutralized.

### Statistical methods

#### Sample size calculation

A retrospective pilot study[16] conducted in the geriatric department of AZ Alma during the 2018 influenza season reported a 7.7% mortality rate among 541 admissions. We calculated that the prevalence (pre-test probability) for Type 1–3 event was approximately 0.005 per patient-day. Assuming a mean hospital stay of about 10 days, we calculated that at least 500 patients would be required to observe a minimum of 25 such events. The target of a minimum of 25 events represented a balance between capturing enough events to achieve generalizable results and maintaining the study’s feasibility. In our study, 31 Type 1–3 events were observed.

#### Statistical analysis

Statistical analyses were performed using SPSS Statistics version 28 and R version 4.4. Categorical variables are reported as numbers and percentages, while continuous variables as means ± standard deviation (SD) for normal distributions or medians with interquartile ranges (IQR) for non-normal distributions.

*Missing values of vital parameters* were imputed in two stages. In the first stage, missing values were imputed as 0 if the observation met one of the following criteria: (1) it was the first recorded observation for the patient, (2) no prior values had been documented, (3) it was registered more than 12 h after the previous observation, or (4) it was a neutralized observation. In the second stage, any remaining missing value was imputed using the last observation carried forward method, whereby missing scores are replaced with the most recently recorded value.

*Performance comparisons between GEWS and NEWS* were conducted using a multitude of performance metrics, including sensitivity, specificity, positive predictive value (PPV), negative predictive value (NPV), and the receiver operating characteristic curve (ROC), along with the area under the ROC curve (AUROC). Corresponding 95% confidence intervals were calculated using a clustered bootstrap percentile approach with 10.000 bootstrap samples. Statistical significance was determined using a *p* value of < 0.00051 (using Bonferroni correction for multiple testing). However, since the pretest probability of an event Type 1–3 was 0.005 per patient day in our feasibility pilot [16], we chose to follow the methodology of Romero-Brufau et al. [21]. They highlighted that in such cases, additional performance metrics such as precision-recall (PR) curves (PPV versus sensitivity), the area under the precision-recall curve (PR-AUC) and the number-needed-to-evaluate (NNE) may be used to assess predictive accuracy. Since the second objective of our study is to select a threshold with the best balance between performance and clinical burden, we decided to use the methodology of Pankhurst et al. [22]. They calculated the NNE, the rate-of-alarms (ROA) (i.e. the number of alarms per 100 patient-days), and the alerted-outcome-event-rate (AOER) (i.e. the number of alerted outcome events per 100 patient-days) for different threshold responses for NEWS2 to assess the clinical burden. Additionally, differences between EWS performance estimates were evaluated against an empirical null distribution, with *p* values calculated using a Monte Carlo permutation approach with 9999 random permutations.

## Results

### Characteristics of study population and clinical event types

A total of 511 patients were included in the study, with a median age of 85 years (IQR: 81–89). The majority were female (60%). Most patients were clinically frail, with a median CFS of 6 (IQR: 5–7). DNR status was documented for all patients. The median hospital stay was 11 days (IQR: 7–15). An overview of participants’ demographic and clinical characteristics is presented in Table 2. A more detailed overview per hospital is provided in the supplementary materials (addendum 1a). Overall, the study population closely reflects the typical patient demographic of acute geriatric wards in Flanders, Belgium. Delirium was identified in only 8.2% of patients at the time of an event. Moreover, detection rates varied significantly between the three study centers (10.2%, 2.5%, and 12.5%, respectively, addendum 1a).

**Table 2:** Characteristics of study population and clinical events

Characteristics	Median, IQR/n (percentage)
Age (years)	85 (81–89)
Sex (women)	307 (60%)
Length of hospital stay (days)	11.8 (7–15)
DNR code - DNR 0 - DNR 1 - DNR 2	511 (100%) 230 (45%) 87 (17%) 194 (38%)
CFS - CFS 3,4,5 - CFS 6 - CFS 7,8,9	6 (4–8)/449 (88%) 138 (27%) 157 (31%) 154 (30%)
Delirium diagnosis (CAM/DOS)	42 (8.2%)
**Clinical event type**	**n (percentage)**
All events (Type 1 – 4) - Type 1 - Type 2 - Type 3 - Type 1 – 3 - Type 4 Surgical intervention Medical intervention	348 7 (2.1%) 12 (3.4%) 12 (3.4%) 31 (8.9%) 317 (91.1%) 8 (2.3%) 309 (88.8%)

IQR: interquartile range; n: number; DNR: Do-Not-Resuscitate code (ranging from code 0 (no restrictions regarding resuscitation or ICU admission) – code 1 (refrain from resuscitation, but ICU admission for invasive ventilation) to code 2 (no resuscitation, nor ICU admission); CFS: Clinical Frailty Scale (ranging from 3 to 9); CAM: confusion assessment method; DOS: delirium observation scale

Table 2 also presents the incidence and classification of clinical events (Type 1–4). A total of 348 clinical events were recorded among 297 patients. Of these events, 317 (91%) were classified as Type 4, usually requiring urgent medical intervention by a geriatrician. Surgical interventions were rare. Type 1, 2 or 3 events occurred in 9%. Further details on patient characteristics and missed inclusions by hospital, the interventions administered for Type 4 clinical events, and the distribution of vital parameters (median, IQR) during events and non-events, are reported in Addendum 1a-c. In addition, baseline characteristics and event rates for the derivation (development and feasibility pilot for Event type 1–2) and validation cohorts are presented in Addendum 1d.

### Validation of GEWS vs. NEWS

**Fig. 1:**
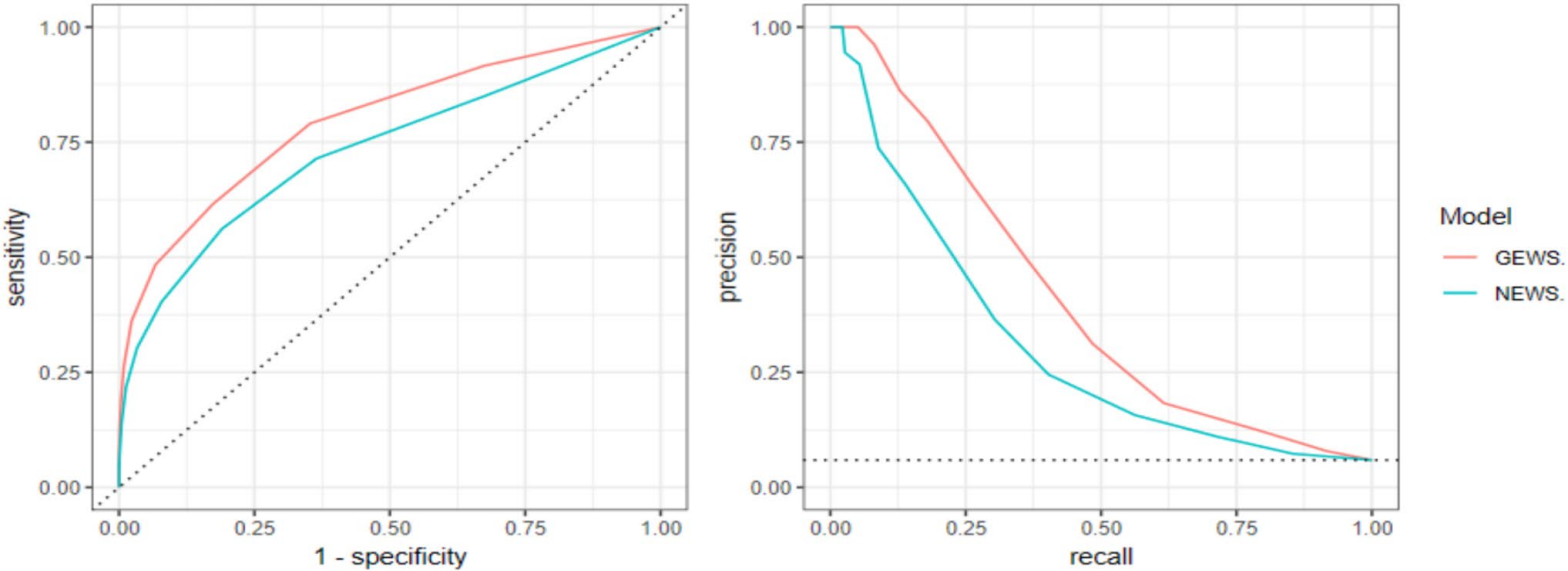
Receiver operating characteristic curve (ROC-curve) and precision-recall curve (PR-curve) for GEWS and NEWS, all clinical events (Type 1 – 4). Left panel: ROC-curve for GEWS (red line) versus NEWS (blue line); Right panel: PR-curve for GEWS (red line) and NEWS (blue line); dotted lines represent performance levels expected by chance alone

The AUROC for GEWS was 0.796 [95% CI 0.770–0.822] compared to 0.732 [95% CI 0.697–0.765] for NEWS (*p* < 0.0001). Similarly, the PR-AUC for GEWS was 0.412 [95% CI 0.350–0.472], whereas for NEWS it was 0.305 [95% CI 0.249–0.365], (*p* < 0.0001) (Fig. 1).

In line with most EWS studies that focus on life-threatening events only, we also evaluated the AUROC and PR-AUC for the subset of Type 1–3 clinical events. In this subgroup, no statistically significant differences were observed between GEWS and NEWS (AUROC: *p* = 0.24; PR-AUC: *p* = 0.995; Addendum 2).

### Determination of the optimal GEWS threshold for all clinical events (Type 1–4)

#### Statistical performance

Performance metrics for NEWS ≥ 5 S3 and GEWS ≥ 4 S3 thresholds across all clinical events (Type 1–4) are summarized in Table 3.

**Table 3:** Performance metrics comparison of NEWS ≥ 5 S3 vs. GEWS ≥ 4 S3 and GEWS ≥ 5 S3, respectively, for all clinical events (Types 1 – 4)

Metrics	All clinical events (Type 1 – 4), (n = 348)				
	NEWS ≥ 5 S3	GEWS ≥ 4 S3	*p* value	GEWS ≥ 5 S3	*p* value
Alerts (n)	879	1291		773	
Accuracy	0.900	0.881	< *0.0001*	0.915	** < 0.0001**
NPV	0.958	0.967	** < 0.0001**	0.961	0.0085
PPV	0.256	0.253	0.8151	0.323	** < 0.0001**
Sensitivity	0.357	0.519	** < 0.0001**	0.397	0.0391
Specificity	0.934	0.903	< *0.0001*	0.948	** < 0.0001**
ROA	17.055	25.049	< *0.0001*	14.998	0.009
NNE	3.907	3.948	0.8151	3.092	** < 0.0001**
AOER	4.366	6.345	< *0.0001*	4.851	0.032

n: number; NPV: negative predictive value; PPV: positive predictive value; ROA: rate of alarms; NNE: number-needed-to-evaluate; AOER: alerted outcome event rate; NEWS: national early warning score; GEWS: geriatric early warning score; (S3): single score of 3 on any vital parameter. p value in **bold** favors GEWS, p value in *italics* favors NEWS

GEWS ≥ 4 S3 demonstrated significantly higher NPV and sensitivity compared to NEWS ≥ 5 S3, (*p’s* < 0.0001). However, GEWS ≥ 4 S3 had a significantly lower accuracy and specificity than NEWS ≥ 5 S3, (*p’s* < 0.0001), with no difference in NNE, but with significantly higher ROA and AOER (*p’s* < 0.0001). These findings raised concerns regarding potential increases in clinical burden on nurses and doctors, alarm fatigue and reduced adherence to EWS protocols.

We therefore examined an alternative threshold: GEWS ≥ 5 S3 (also presented in Table 3). At similar NPV and sensitivity levels, GEWS ≥ 5 S3 showed significantly higher accuracy, PPV and specificity compared to NEWS ≥ 5 S3, (*all p’s* < 0.0001). Since only 5 out of 297 observations with an S3 score, were followed by a clinical event (Type 1–4), we also evaluated the same thresholds excluding S3 (Table 4). Exclusion of S3 had minimal impact on the NPV for both NEWS and GEWS. Across all performance metrics GEWS ≥ 5 continued to outperform NEWS ≥ 5.

**Table 4:** Performance metrics comparison of GEWS ≥ 5 vs. NEWS ≥ 5 excluding the S3 score, for all clinical events (Type 1 – 4)

Metrics	All clinical events (Type 1 – 4), (n = 348)		
	NEWS ≥ 5	GEWS ≥ 5	*P* value
Alerts (n)	523	459	
Accuracy	0.927	0.940	** < 0.0001**
NPV	0.956	0.960	** < 0.0001**
PPV	0.365	0.497	** < 0.0001**
Sensitivity	0.303	0.362	** < 0.0005**
Specificity	0.967	0.977	** < 0.0001**
ROA	10.147	8.906	0.0029
NNE	2.738	2.013	** < 0.0001**
AOER	3.706	4.424	** < 0.0005**

n: number; NPV: negative predictive value; PPV: positive predictive value; ROA: rate of alarms; NNE: number-needed-to-evaluate; AOER: alerted outcome event rate; NEWS: national early warning score; GEWS: geriatric early warning score; (S3): single score of 3 of any vital parameter. p value in bold favors GEWS

#### Clinical burden

**Fig. 2:**
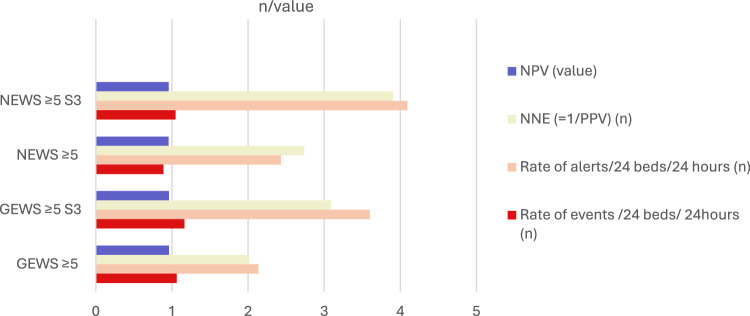
Clinical burden of NEWS and GEWS at different thresholds, with and without the S3 score, across all clinical events (Type 1 – 4), during a 24-h period in a 24-bed acute geriatric ward. NNE: number-needed-to-evaluate; PPV: positive predictive value; ROA: rate-of-alarms; AOER: alerted- outcome-event-rate; h: hours; NEWS: national early warning score; S3: single score of 3 on any vital parameter; GEWS: geriatric early warning score;* p* value in bold favors GEWS

From a clinical workload perspective, the NNE, the ROA and the AOER are particularly relevant. These measures are illustrated in Fig. 2 and its accompanying table.

Among the evaluated thresholds, GEWS ≥ 5 provided the best overall balance between predictive value and workload, particularly with respect to NPV, NNE and ROA. Although GEWS ≥ 5 *with* S3 demonstrated a better AOER, with an improvement of 0.102 events per 24 h in a 24-bed ward compared to GEWS ≥ 5 alone, this came at the cost of a substantially higher ROA – with an increase of 1.463 alerts. Practically, detecting one additional true event over a 10-day period using GEWS ≥ 5 *with* S3 would generate approximately 14 alarms, 13 of which would be false positives.

### Determination of the optimal GEWS threshold for life-threatening events (Type 1–3)

#### Statistical performance

For life-threatening events (Type 1–3), the differences in AUROC and PR-AUC between NEWS and GEWS were not statistically significant (Addendum 2).

#### Clinical burden

From a clinical workload perspective, GEWS ≥ 8 showed significantly higher specificity (0.999 vs. 0.995, *p* < 0.0001) and a substantially lower ROA (0.518 vs. 1.347, *p* < 0.0001) compared with NEWS ≥ 7, without a significant difference in AOER (0.249 vs. 0.415, *p* = 0.0226) (Addendum 3). Notably, with GEWS ≥ 8, approximately one in every two alarms corresponded to a true life-threatening event, compared to one in every 2.5 for NEWS ≥ 7. Practically, NEWS ≥ 7 would trigger about three additional alarms over 25 days to detect one additional true life-threatening event, with two alarms being false positive.

## Discussion

Currently used EWSs, such as NEWS and MEWS, have limited accuracy in predicting clinical deterioration in frail older patients [8–10, 22]. In this study, we validated a specifically developed GEWS against NEWS and compared their performance metrics for detecting clinical deterioration in frail older patients admitted to the ED and the acute geriatric ward. In addition, we determined the GEWS threshold that offers the best balance between predictive accuracy and clinical feasibility, with the goal of supporting safer and more efficient care delivery in acute geriatric settings.

Our results indicate that GEWS significantly outperforms NEWS in identifying clinical deterioration in frail older patients across all clinical events (Type 1–4). This was demonstrated by a significantly higher AUROC and PR-AUC compared to NEWS. For life-threatening events in-hospital (Type 1–3), GEWS performed comparably to NEWS with no statistically significant differences in AUROC or PR-AUC, supporting a non-inferiority of GEWS in this context (addendum 2).

These findings contribute to the limited available literature evaluating the performance of existing EWSs in detecting clinical deterioration in frail older patients. Churpek et al. [8] previously reported that the AUC of MEWS for detecting cardiac arrest was significantly lower in older hospitalized patients compared to non-older patients (AUC 0.71 (95% CI [0.68–0.75]) vs. 0.85 (95% CI [0.82–0.88]), *p* < 0.001). Likewise, Mitsunaga et al. [23] assessed NEWS and MEWS in the ED for predicting in-hospital mortality in older patients and found an AUC of 0.789 (95% CI [0.747–0.829]) for NEWS and 0.720 (95% CI [0.671–0.765]) for MEWS. More recently, IEWS achieved an AUROC of 0.77 (95% CI [0.73–0.81]) for predicting in-hospital mortality among patients over 80 years [13]. The FaP-ED validation study on 30-day mortality showed that combining frailty (mean CFS = 4) with NEWS improved performance, yielding an AUROC of 0.86 (95% CI [0.83–0.90]) [12]. In our study (median 85y and CFS 6), the AUROC of GEWS for life-threatening clinical events (Type 1–3) was comparable, at 0.847 [95% CI 0.779–0.905], and higher than that of NEWS (0.806 [95% CI 0.735–0.882]). However, this difference did not reach statistical significance (*p* = 0.24).

Although direct comparisons of our results with the aforementioned studies are not possible due to different patient characteristics and study design, our findings suggest that GEWS may represent a more accurate tool for predicting clinical deterioration in frail older, hospitalized patients, particularly when considering all clinical events (Type 1–4).

We first evaluated the performance of GEWS at the predefined threshold of ≥ 4 S3 for all clinical events (Type 1–4). Haegdorens et al. [24] emphasized that EWSs are primarily designed to effectively *rule out* serious adverse events and, therefore, should prioritize a high NPV. In this regard, GEWS ≥ 4 S3 showed a significantly better NPV than NEWS ≥ 5 S3.

However, as GEWS ≥ 4 S3 captured more clinical events (higher AOER) than GEWS ≥ 5 S3, it generated nearly 25% more alarms (ROA), substantially increasing the clinical burden. When the objective shifts from ruling out to *ruling in* patients requiring escalation of care, a high PPV becomes essential. At this level, GEWS ≥ 5 S3 outperformed NEWS ≥ 5 S3, with a significantly higher PPV and lower NNE, while maintaining a comparable NPV and AOER. Moreover, GEWS ≥ 5 S3 tended to produce fewer alarms (ROA, *p* = 0.009). Interestingly, excluding the S3 component from GEWS ≥ 5 further improved PPV, and reduced both ROA and NNE. Taken together, across all clinical events (Type 1–4), GEWS ≥ 5 *without S3* offers the best balance between predictive performance and clinical burden, making it the preferred threshold for predicting clinical deterioration in older patients with frailty in the Belgian context.

Finally, since the Royal College of Physicians recommends that a NEWS ≥ 7 should trigger an urgent high-level clinical response, we evaluated the predefined GEWS threshold of ≥ 8 for identifying life-threatening events (Type 1–3) (see addendum 3). Compared to NEWS ≥ 7, GEWS ≥ 8 showed significantly higher specificity and lower ROA, as well as the lowest NNE, while maintaining a comparable NPV. Given the high resource demands associated with these interventions, a threshold of GEWS ≥ 8 appears more appropriate for triggering such responses. In contrast, GEWS ≥ 5 may be better suited for triggering alarms to be managed by the attending ward physician if available. This corresponds with the tiered system that exists for the NEWS (2).

Irrespective of the threshold applied, the clinical judgment of nurses should not be ignored. Previous studies have shown that combining EWS with nurses’ clinical assessment yielded non-inferior accuracy [25], and that combining the Nurse Intuition Patient Deterioration Scale (NIPDS) with NEWS provided greater net benefit [26]. Moreover, preserving and strengthening nurses’ critical thinking and clinical judgement skills remains essential to ensure that EWSs support, rather than replace, professional expertise [27].

### Practical recommendations for using the GEWS

A GEWS ≥ 5 threshold is recommended as the standard trigger for nursing staff in the ED and acute geriatric ward to notify the geriatrician for all clinical event Types (1–4). To address concerns regarding sensitivity and AOER, the following additional steps are advised: (1) When GEWS equals 4, nurses closely monitor the patient with a reassessment within 2 h. If any further deterioration is observed, the attending physician should be promptly alerted; (2) Any S3 on a vital parameter should prompt an immediate recheck of all vital signs to confirm or rule out transient abnormalities, reassessment is mandated; (3) For GEWS ≥ 8, an urgent response by staff with critical care expertise should be initiated, including a prompt bedside assessment, unless it has been previously documented that the patient does not wish to be resuscitated or transferred to an ICU; (4) Lastly, and most importantly, if a nurse has a strong clinical suspicion or ‘gut feeling’ of deterioration, appropriate action should be taken irrespective of the GEWS score.

### Strength and limitations

First, we achieved our goal of validating a GEWS specifically tailored to frail older adults, using vital parameter cut-off values adjusted to their physiological characteristics. Second, unlike conventional EWSs, which primarily aim to prevent ICU transfers or cardiopulmonary resuscitation, GEWS focuses on patient-centered outcomes, including Type 4 clinical events. Third, our inclusion policy avoided bias related to ageism: patients for whom ICU-admission was no longer appropriate were still represented through Type 4 clinical events. Additionally, in contrast to other previous studies, patients unable to provide informed consent because of cognitive impairment were included via caregiver consent, while those in palliative care settings—where interventions were considered inappropriate—were excluded. Fourth, our study demonstrates that GEWS is applicable both in the ED and on the geriatric ward. Considering these aspects, our study aligns with the clinicals needs of frail older patients, enabling more proactive care in alignment with the patients’ preferences. A fifth strength is the study’s real-world multicenter design, conducted without additional training for nurses or geriatricians. This enhances the external validity of the findings – at least within Belgian geriatric wards and emergency departments. None of the participating centers employed high level medical assistance teams linked to NEWS or GEWS scores. Instead, nurses acted based on clinical judgement or clinical management plans.

A sixth strength is the use of robust methodology, not only including C-statistics, but also performance metrics such as PR-AUC, alongside an analysis of clinical burden at different thresholds. This comprehensive evaluation provides a more nuanced understanding of GEWS performance, with potential to reduce false-positive alarms, mitigate alarm fatigue and improve adherence to the GEWS protocol. Our approach is consistent with best practices for EWS evaluation and aligns with the methodology applied by Pankhurst et al.[22] in assessing NEWS2 response thresholds in a UK acute hospital setting.

Nonetheless, several limitations should be acknowledged. First, the study population included no robust older adults (i.e. CFS < 3), limiting the applicability of the findings to that subgroup. Since robust and frail older individuals may differ physiologically, NEWS may remain more appropriate for robust patients until further comparative studies are available.

Second, the classification of Type 4 clinical events was based on a predefined list of medical interventions, which in hindsight may have led to an underestimation of clinical event rates. Notably, the administration of intravenous analgesia for severe pain (NRS ≥ 7) was not included as a qualifying Type 4 event. While this omission was unintentional, it may have impacted the measured GEWS performance by overlooking relevant acute clinical responses.

Third, we did not calculate an AUC or assess calibration in our feasibility pilot study to determine the original predictive performance in the derivation cohort. We were confident about the original cut-off values for vital parameters used in the GEWS, since they were proven appropriate for our clinical context.

Fourth, no item-level analysis was conducted to assess the individual contribution of each vital parameter to the overall GEWS performance. Some parameters may have had limited impact. For instance, respiratory rate – despite being a highly sensitive indicator of deterioration – was often estimated rather than measured, potentially reducing GEWS sensitivity. Improving awareness through training, along with the implementation of validated automated monitoring tools for older adults, could enhance sensitivity. Furthermore, pain was not consistently recorded alongside other vital parameters in two of the three centers, resulting in a higher rate of missing data (see addendum 1c). Moreover, literature remains inconclusive about the prognostic value of pain as a sixth vital parameter for clinical deterioration, although clinical experience suggests it can be an early indicator in frail older patients potentially by increasing cardiovascular stress. At last, delirium was identified in only 8.2% of patients based on CAM or DOS assessments – substantially lower than the expected prevalence of 23.0% [28]. This under-detection likely reflects reduced use of these screening tools during routine care, which was in part due to disruptions in nursing education and clinical routines during the COVID-19 pandemic. CAM was perceived as complex to administrate, while DOS requires three observations and annual refresher training, which were not consistently maintained during the study period. As a result, the true prevalence of delirium in our study population was likely under-estimated, limiting our ability to evaluate how well GEWS – via the AVPU scale – captures delirium-related deterioration.

A fifth limitation concerns the potential impact of the so-called “treatment paradox,” in which initiating treatment may prevent a more severe event and thereby lead to an underestimation of an EWS’s accuracy. In our study, we reduced the potential bias by evaluating physiological parameters up to 12 h prior to each event and neutralizing parameters after each event. Also, explicit inclusion of the treatment given to avoid a Type 1–3 event by classifying this as a Type 4 event improved the prognostic performance [29]. Only in the case that a Type 4 event was followed more than 12 h later by a Type 1–3 event, the therapeutic intervention could have influenced the parameters of the Type 1–3 event*.* However, this influence is comparable with EWS studies that do not take Type 4 events into account. Nevertheless, minor influence cannot be excluded for patients who received pre-hospital treatment.

Finally, although this was a multicenter study, the findings may be context-dependent and influenced by characteristics of the Belgian healthcare system, including relatively low nurse-to-patient ratio compared with other European settings [30]. As such, generalizability to other healthcare settings with different staffing levels, electronic patient record systems, or clinical escalation protocols may be limited. In care settings where nurse-to-patient ratios are higher and rapid response capacity is greater, it may be reasonable to adopt a GEWS threshold of ≥ 4 S3, accepting a higher clinical burden in exchange for the highest AOER.

### Proposed future research

To further refine GEWS, efforts should focus on increasing its PPV while reducing clinical burden and alarm fatigue. A promising direction is the integration of structured clinical intuition, given the recognized impact of nurse intuition on patient outcomes [31]. Haegdorens et al. proposed the NIPDS to quantify such intuition [31]. A Belgian study conducted on medical, surgical and geriatric wards found that combining NIPDS with NEWS improved the prediction of severe adverse events [26]. However, as NIPDS is only validated for the first 24 h of admission, its utility in longer hospital stays remains unclear. Moreover, some NIPDS features may already be indirectly captured by GEWS. For example, while older adults may underreport pain, NIPDS captures it through facial cues – yet GEWS already includes structured pain assessment (NRS or PAINAD), unlike NEWS. NIPDS also tracks behavior and responsiveness to detect consciousness changes, which GEWS evaluates more gradually than NEWS. These overlaps suggest limited added value from integrating NIPDS into GEWS without further validation.

Another key priority is improving the detection of delirium, especially given its prognostic importance and its role as an early marker of acute deterioration in frail older adults. We propose that future studies consider evaluating the Richmond Agitation and Sedation Scale (RASS) as an alternative to AVPU within GEWS. RASS provides a graded assessment of consciousness through direct observation and has demonstrated high sensitivity (82.0%) and specificity (85.1%) for identifying delirium in older ED patients [32]. Furthermore, changes in RASS have been associated with improved specificity, up to 92.0% [32]. The scale is time-efficient (requiring less than 10 s), does not depend on proxy input, and is applicable across various clinical environments, including the ED, ICU, acute ward and perioperative settings. However, RASS is not intended to diagnose delirium. Therefore, incorporating a structured approach to delirium screening (e.g. 4AT-score might be still warranted alongside GEWS to diagnose delirium.

Finally, future studies should include an item-level analysis to evaluate the individual predictive contribution of each GEWS parameter to the overall GEWS score performance. Especially, comparing GEWS with and without the inclusion of the pain parameter – measured appropriately using instruments such as the PAINAD or NRS – could help to determine its relevance and impact within the scale.

## Conclusion

This study validated GEWS as a more accurate alternative to NEWS for detecting clinical deterioration within 12 h in older patients with frailty receiving acute geriatric care across all clinical events (Types 1–4). A GEWS threshold of ≥ 5 was found to provide the optimal balance between predictive performance and clinical burden, making it the most appropriate threshold for routine monitoring in a Belgium context. By facilitating earlier and more individualized interventions, GEWS supports care that is better aligned with the complex needs and preferences of older patients with frailty. For triggering more urgent medical assistance, a threshold of GEWS ≥ 8 is recommended. Importantly, clinical judgement of nurses should not be ignored, irrespective of the GEWS score.

## Supplementary Information

Below is the link to the electronic supplementary material.

Supplementary file1 (DOCX 118 KB)

## Data Availability

The data that support the findings of this study are available from the ctc@jessazh.be upon reasonable request.
