# Supplementary material for: Validation and performance of a geriatric early warning score (GEWS) versus the national early warning score (NEWS) in predicting clinical deterioration in frail older patients
Source: Eur Geriatr Med. 2025 Oct 6;17(2):615–27. doi: 10.1007/s41999-025-01316-7 (PMC13109162; doi:10.1007/s41999-025-01316-7)
Supplement: Supplementary file 1 — Supplementary file1 (DOCX 118 KB) [file 41999_2025_1316_MOESM1_ESM.docx]

SUPPLEMENTARY MATERIALS

**Addendum 1**

**1a: Patient characteristics across the three participating hospitals**

| **Characteristics** | **Hospital 1** | **Hospital 2** | **Hospital 3** | **All hospitals** |
| --- | --- | --- | --- | --- |
| **Hospital Type** | Regional teaching | Regional  teaching | Supra-regional teaching |  |
| **Hospital beds** (n) | 431 | 310 | 981 |  |
| **Acute geriatric care beds** (n) | 100 | 72 | 149 |  |
| **Participating beds** | 70 | 72 | 30 | 172 |
| **Admissions during inclusion period in participating beds (n)** | 477 | 337 | 199 | 1013 |
| **Geriatricians including patients (n)** | 4 | 1 | 1 | 6 |
| **Patients included** (n,% of admission) | 274 (57%) | 157 (47%) | 80 (40%) | 511 (50%) |
| **All clinical events** (n) | 225 | 84 | 39 | 348 |
| **Age** (years) (median, IQR) | 85 (82-90) | 86 (80-88) | 86 (81-89) | 85 (81-89) |
| **Sex** (female) (n, %) | 164 (59.8%) | 88 (56.1%) | 55 (68.8%) | 307 (60.1%) |
| **Length of hospital stay** (days) (median, IQR) | 11 (8-16) | 9 (6-14) | 10 (7-15) | 11 (7-15) |
|  |  |  |  |  |
| **DNR coding present (n,%)**   - DNR 0 - DNR 1 - DNR 2 | 274 (100%)  131 (47.8%)  42 (15.3%)  101 (36.9%) | 157 (100%)  66 (42.0%)  35 (22.3%) 56 (35.7%) | 80 (100%)  33 (41.2%)  10 (12.5%)  37 (46.3%) | 511 (100%)  230 (45.0%)  87 (17.0%)  194 (38.0%) |
| **CFS code present (n, %)**   - CFS 3-5 - CFS 6 - CFS 7-9 | 212 (77.3%)  74 (27.0%)  64 (23.3%)  74 (27.0%) | 157 (100%)  45 (28.7%)  57 (36.3%)  55 (35.0%) | 80 (100%)  19 (23.8%)  36 (45.0%)  25 (31.2%) | 449 (87.8%)  138 (27.0%)  157 (30.7%)  154 (30.1%) |
| **Delirium diagnosis (n, %)** | 28 (10.2%) | 4 (2.5%) | 10 (12.5%) | 42 (8.2%) |
| **All events (n)**   - Event type 1 (n) - Event type 2 (n) - Event type 3 (n) - Event type 1-3(n) - Event type 4 (n)   - Surgical   - Medical   intervention | 225  2  9  10  21  204  3  201 | 84  3  2  2  7  77  3  74 | 39  2  1  0  3  36  2  34 | 348  7  12  12  31  317  8  309 |

_n: number; IQR: interquartile range; %: percentage; DNR: Do-Not-Resuscitate code (ranging from code 0 (no restrictions regarding resuscitation or ICU admission) – code 1 (refrain from resuscitation, but ICU admission for invasive ventilation) to code 2 (no resuscitation, nor ICU admission); CFS: Clinical Frailty Scale (ranging from 3-9, 62 missing values)._

**1b : Details on administered medical interventions for Type 4 clinical events**

| **Medical interventions** | **Episode 1** | **Episode 2** | **Total** |
| --- | --- | --- | --- |
| **Consciousness related interventions:** |  |  |  |
| Intravenous (IV) glucose (> 6 g) for acute hypoglycemic coma | 3 | 0 | 3 |
| **Cardiovascular interventions:** |  |  |  |
| IV diuretics (for acute pulmonary oedema) | 41 | 11 | 52 |
| IV or oral anti-arrhythmics (for life-threatening arrhythmias) | 8 | 3 | 11 |
| IV nitrates (for unstable angina pectoris) | 3 | 0 | 3 |
| **Bleeding or hemostasis interventions:** |  |  |  |
| Transfusion of packed red blood cells, platelets (for massive hemorrhage) | 15 | 4 | 19 |
| Synthetic plasma factors (for massive hemorrhage) | 3 | 0 | 3 |
| Anticoagulants (e.g. low-molecular-weight heparin (LMWH)), heparin,  thrombolytics (for embolism, stroke or myocardial infarction) | 13 | 0 | 13 |
| **Sepsis and dehydration related interventions:** |  |  |  |
| Volume resuscitation with fluid challenge (crystalloids/electrolytes/albumin) | 133 | 13 | 146 |
| IV or oral antibiotics (oral quinolones) | 156 | 20 | 176 |
| IV corticosteroids (for acute Addison’s disease)^2^ | 26 | 3 | 29 |
| **Respiratory interventions:** |  |  |  |
| Oxygen supplementation : Augmentation by ≥ 100 % | 53 | 10 | 63 |
| IV corticosteroids (for COPD exacerbation)^2^ | 26 | 3 | 29 |
| **Total medical interventions type 4 events** | **454** | **64** | **518** |

_The hospitalization period was divided into two episodes._ **_Episode 1_** _started upon admission to the emergency department or the acute geriatric ward._ **_Episode 2_** _began either 72 hours after admission – if no clinical event occurred during the first phase – or 72 hours after the last clinical event recorded within episode 1._ ^2^ _No differentiation between purpose for iv corticoids was documented, therefore both possibilities are mentioned._

**1c:** **Median (IQR) or number (percentage)** **of vital parameters (Non-Event versus Event); and corresponding missing values**

| **Vital parameter** | **Non-Event**, N = 9,977*^1^* | **Event**, N = 630*^1^* |
| --- | --- | --- |
| Respiration rate (p/m) | 18.00 (14.00-18.00) | 18.00 (14.00-20.00) |
| Missing values | 653 | 134 |
| Oxygen Saturation (%) | 96.00 (95.00-98.00) | 96.00 (93.00-98.00) |
| Missing values | 474 | 52 |
| Oxygen therapy (l/min) | 0.00 (0.00-0.00) | 1.00 (0.00-2.00) |
| Missing values | 3,740 | 231 |
| Body temperature (°C)^2^ | 36.20 (36.00-36.50) | 36.60 (36.20-37.30) |
| Missing values | 577 | 112 |
| SBP (mmHg) | 136 (119-154) | 132 (113-152) |
| Missing values | 341 | 71 |
| Heart rate (bpm) | 74 (65-83) | 80 (69-93) |
| Missing values | 308 | 59 |
| Level of consciousness (AVPU) |  |  |
| Alert | 5,260 (100%) | 92 (94%) |
| Voice stimulus | 0 (0%) | 4 (4.1%) |
| Pain stimulus | 5 (<0.1%) | 1 (1.0%) |
| Unresponsive/Agitation | 3 (<0.1%) | 1 (1.0%) |
| Missing values | 4,709 | 532 |
| Pain (NRS/PAINAD) | 0.00 (0.00-0.00) | 0.00 (0.00-2.00) |
| Missing values | 2,024 | 303 |

*^1^*  _IQR: interquartile range; SD: standard deviation; n: number; %: percentage p/m: per minute; L/min: liter per minute; °C: degree Celsius,_ ^2^ _temperature measured at the axilla, temporal or tympanic sites. For forehead measurements, 0.2°C was added to the recorded value; SBP: systolic blood pressure; mmHg: millimeters of mercury; bpm: beats per minute; NRS: Numeric rating scale (ranging from 0-10); PAINAD: Pain in advanced dementia scale (ranging from 0-10)_

**Addendum 1d: *Baseline Characteristics and event rates for the derivation vs. validation cohort***

|  | Development GEWS | Feasibility pilot study 2018 | Validation Cohort 2022 |
| --- | --- | --- | --- |
| Population Characteristics | Typical cohort hospitalized on acute geriatric ward (CCI > 4 in 52% patients, 80 % of patients using a walking aid, average UGS 0.33m/sec, mean age 85 y) | Typical cohort hospitalized on acute geriatric ward  n=14 patients with Type 1 or 2 event: median 86 y, median CCI 9, delirium 71 %, female 36% | Median 85 year, 60.1 % female, CFS 6 |
| Number of patients | N/A | 541 | 511 |
| Number of hospitals | 2 | 1 | 3 |
| Clinical events occurring in (% of patients) (n) | Retrospective | Retrospective | Prospective |
| Clinical events type 1-3 | 9 % (estimated) | 42 | 31 |
| Clinical events type 3 |  | 28^1^ | 12^2^ |
| Clinical events type 1-2 |  | 14 | 19 |
| Clinical events type 4 | 60 % (estimated) | N/A | 317 |

_CCI: Charlson Co-morbidity Index; UGS: Usual Gait Speed; CFS: Clinical Frailty Scale; GEWS: Geriatric early warning score; MEWS: Modified early warning score; ED: Emergency Department; n.i. : not investigated_ ^1^ _all palliative patients included;_ ^2^ _palliative patients at the start not included;_

**Addendum 2**

**The receiver operating curve (ROC) and precision-recall curve (PR) for GEWS and NEWS, severe clinical events (Type 1 – 3).**

**
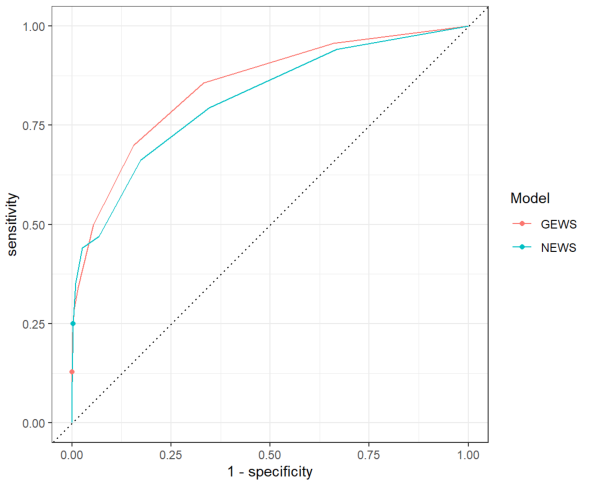

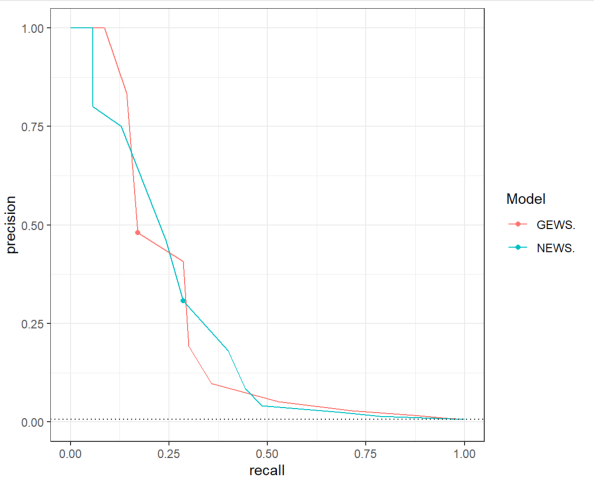
**

**Left panel**: ROC-curve for GEWS (red line) versus NEWS (blue line); **Right panel**: PR-curve for GEWS (red line) and NEWS (blue line); dotted lines represent performance levels expected by chance alone.

The AUROC for GEWS was 0.847 [95% CI, 0.779-0.905], compared to 0.806 [95% CI, 0.735-0.882] for NEWS, *(p*=0.24*)*. The PR-AUC was nearly identical between GEWS (0.246 [95% CI, 0.123-0.394]) and NEWS (0.245 [95% CI, 0.113-0.393]), (*p*=0.995)

**Addendum 3**

**Performance metrics of GEWS ≥8 vs. NEWS ≥7, for clinical events Type 1 – 3**

|  | **Clinical events Type 1 – 3 (n=31)** | | |
| --- | --- | --- | --- |
| **Metrics** | **NEWS ≥7** | **GEWS ≥8** | ***p*-value** |
| **Alerts (n)** | 65 | 25 |  |
| **Accuracy** | 0.991 | 0.993 | 0.0042 |
| **NPV** | 0.995 | 0.994 | 0.0226 |
| **PPV** | 0.308 | 0.480 | 0.0222 |
| **Sensitivity** | 0.286 | 0.171 | 0.0226 |
| **Specificity** | 0.995 | 0.999 | **<0.0001** |
| **ROA** | 1.347 | 0.518 | **<0.0001** |
| **NNE** | 3.250 | 2.083 | 0.0222 |
| **AOER** | 0.415 | 0.249 | 0.0226 |

_n: number; NPV: negative predictive value; PPV: positive predictive value; ROA: rate of alarms; NNE: number needed to evaluate; AOER: alarmed outcome event rate; NEWS: national early warning score; GEWS: geriatric early warning score;_ *_p_* _-value in_ **_bold_** _favors GEWS._
